# Mortality associated with *Dictyocaulus cervi* in farmed red deer (*Cervus elaphus*) in Romania

**DOI:** 10.1007/s00436-026-08641-1

**Published:** 2026-02-06

**Authors:** Andrada-Silvia Cârstolovean, Andrada Negoescu, Marian Taulescu, Cristina Daniela Cazan, Flaviu Alexandru Tăbăran, Raluca Marica, Andrei Paul Ungur, Călin Mircea Gherman, Andrei Daniel Mihalca

**Affiliations:** 1 Department of Parasitology and Parasitic Diseases, University of Agricultural Sciences and Veterinary Medicine of Cluj-Napoca, 3-5 Calea Mănăștur, Cluj-Napoca, 400372 Romania; 2 Department of Anatomic Pathology, University of Agricultural Sciences and Veterinary Medicine of Cluj-Napoca, 3-5 Calea Mănăștur, Cluj-Napoca, 400372 Romania; 3 Parasitology Consultancy Group, Corușu, Romania

**Keywords:** *Dictyocaulus cervi*, Red deer, Verminous bronchitis, Romania

## Abstract

Lungworms from the genus *Dictyocaulus* are known to cause catarrhal bronchitis and pulmonary atelectasis in domestic and wild ruminants. In red deer (*Cervus elaphus*), four *Dictyocaulus* species can be present in the respiratory tract. This study describes the investigation of mortalities in red deer (*Cervus elaphus*) in a commercial farm for red deer from Romania, associated with the presence of lungworms. Postmortem examination revealed the presence of nematodes in the bronchial lumen in all cases. Morphological and molecular examination of the nematodes recovered confirmed *Dictyocaulus cervi* as the species involved. The most probable cause of death in all four cases was respiratory failure due to the severe parasitic infection and cachexia. This study represents the first report of mortality associated with *Dictyocaulus cervi* in farmed deer and the first report of the species in Romania, highlighting the need for further epidemiological studies to assess its distribution and potential impact on other cervids in the country.

**Supplementary Information:**

The online version contains supplementary material available at 10.1007/s00436-026-08641-1.

## Background

Farmed deer are managed for various purposes, including meat production, trade to hunting preserves, and use in agritourism farms. Similar to wild populations of cervids, they are susceptible to a range of pathogens, including parasites. Among these, liver flukes, gastrointestinal strongyles, and lungworms can affect several species of cervids. Lungworms can be classified into two main groups: those with a direct life cycle, primarily species of genus *Dictyocaulus*, and those that require an intermediate host, belonging to the family Protostrongylidae (Haigh et al. 2002; Mattiello 2009).

Several *Dictyocaulus* species can cause parasitic bronchitis in both domestic and wild ruminants. According to the latest morphological and molecular studies, the respiratory tract of red deer (*Cervus elaphus*) can be parasitized by four species of *Dictyocaulus* Railliet & Henry,^1988^, namely *D. viviparus* (Bloch, 1782) Raillet & Henry, 1988, *D. eckerti* Skrjabini, 1931, *D. cervi* Pyziel et al. 2017 *D*. *skrjabini* Pyziel et al. 2023. (Pyziel et al. 2017, 2023; Carreno et al. 2009; Acs et al., 2016, Durette-Desset et al. 1988; Gibbons and Hoglund, 2002, Hoglund et al., 1999, Hugonnet and Cabaret 1987; Gibbons and Khalil 1988).

The pathogenicity of *D. viviparus* and *D. filaria* is well established in cattle and sheep (Deplazes et al. 2016; McCarthy 2019; Stigger et al. 2024), respectively, with recent reports also documenting their presence in wild ruminants (Cârstolovean et al. 2024). Clinical signs in domestic ruminants may include nasal discharge, coughing, pulmonary emphysema, and pneumonia, which are more prevalent in young animals than in adults. In cases of heavy infection, fatal outcomes can occur (Deplazes et al. 2016; McCarthy 2019; Stigger et al. 2024). On the other hand, for some recently described species, such as *Dictyocaulus cervi*, information on their pathogenicity remains limited, and their impact on both free-ranging and farmed cervids is not well understood (Pyziel et al. 2017, 2018).

In this context, we describe a mortality event in a commercial farm for red deer from Romania associated with severe lesions caused by *Dictyocaulus cervi.*

## Materials and methods

Four red deer (*Cervus elaphus*) originating from a commercial farm in Romania (name and location not disclosed for privacy reasons) were submitted to the Department of Veterinary Pathology, University of Agricultural Sciences and Veterinary Medicine of Cluj-Napoca, Romania. The farm had a population of 450 cervids distributed across 16 fenced areas, with the largest one accommodating up to 100 animals. Over the course of one week, April 2024, the four red deer ranging in age from one to six years were found dead.

Postmortem evaluation was performed on four individuals. Multiple tissue samples from the cranial and caudal lobes of the lungs were collected and evaluated by histopathology. They were fixed in 10% buffered neutral formalin and routinely embedded in paraffin. The sections were stained with hematoxylin-eosin (H&E) and assessed using an Olympus BX-42 light microscope. Photomicrographs were captured using an Olympus UC30 digital camera and Stream Basic imaging software (Olympus Corporation, Tokyo, Japan).

Adult nematodes from the bronchial lumen were collected in 5% formalin for morphological identification and in 70% ethanol for molecular confirmation. The morphological identification of nematodes was done using the Olympus microscope (Olympus BX61), based on identification keys and descriptions (Pyziel et al. 2017). Other nematodes were identified based on identification keys and descriptions by Skryabin et al. (1992) and Deplazes et al. (2016). Moreover, Baermann’s method was performed using sections of the lung tissue to show the presence of larvae.

Genomic DNA was extracted from 12 randomly selected adult nematodes (2 males, 11 females) using the ISOLATE II Genomic DNA Kit (Bioline Meridian Bioscience, Luckenwalde, Germany), following the manufacturer’s instructions, and stored at − 20 °C until further use. To analyze the DNA extracts, a conventional Polymerase Chain Reaction (PCR) was performed, targeting the second internal transcribed spacer (ITS-2) of the ribosomal DNA. The PCR reaction mixture included 12.5 µl Green PCR Mastermix (Rovalab GmbH, Teltow, Germany), 6.5 µl ultrapure water, 1 µl (10 pmol/µL) of each primer (NC1: 5′-ACG TCT GGT TCA GGGTTG TT-3′, NC2: 5′-TTA GTT TCT TTT CCT CCG CT-3′) as described by Newton et al. (1998), and 4 µl of the isolated DNA. Amplification was carried out in a Thermal Cycler T1000™ (Bio-Rad, London, UK) using the following conditions: initial denaturation at 95 °C for 5 min, followed by 40 cycles of denaturation at 95 °C for 45 s, annealing at 60 °C for 45 s, and extension at 72 °C for 45 s, with a final extension at 72 °C for 5 min. The PCR products were visualized by electrophoresis on a 1.5% agarose gel stained with ECO Safe 20,000× Nucleic Acid Staining Solution (Pacific Image Electronics, New Taipei, Taiwan), and compared to a molecular weight marker (O’GeneRuler™ 100 bp DNA Ladder, Thermo Fisher Scientific Inc., Waltham, MA, USA). The PCR product was purified using the ISOLATE II PCR and Gel Kit (Bioline Meridian Bioscience, Luckenwalde, Germany) and submitted for sequencing (Macrogen Europe, Amsterdam, The Netherlands). The resulting sequences were analyzed through GenBank™ using the Basic Local Alignment Search Tool (BLAST) and Geneious^®^ 4.85 software (Kearse et al. 2012).

## Results

**Fig. 1:**
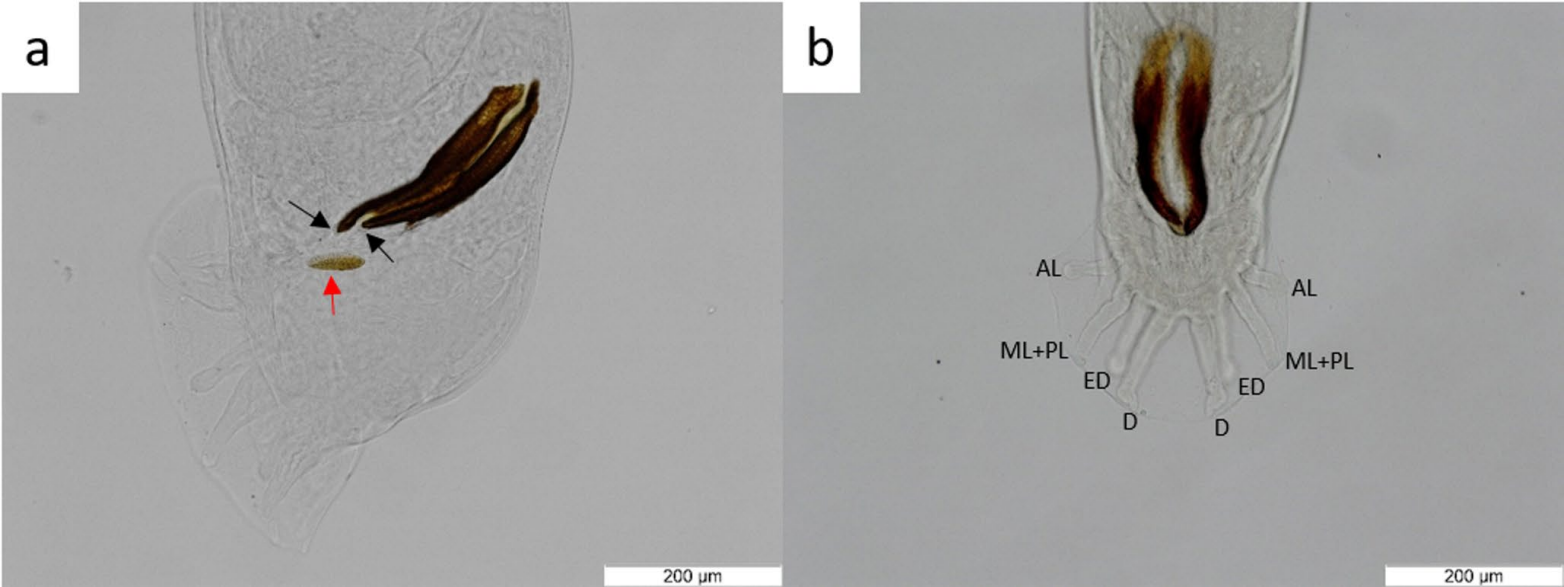
*Dictyocaulus cervi* male posterior end: (**a**) Lateral view of the posterior end with the presence of the two dark brown spicules, with their tip surrounded by a transparent membrane (black arrows), and the gubernaculum (red arrow). (**b**) Dorsal view of the posterior end, with the specific morphology of the rays: anterolateral (**AL**) with rounded distal tip, mediolateral (**ML**) and posterolateral (**PL**) completely fused, externodorsal (**ED**) shorter than the dorsal rays and with rounded distal tip, dorsal (**D**) with the presence of two or three small divisions at distal tip

**Fig. 2:**
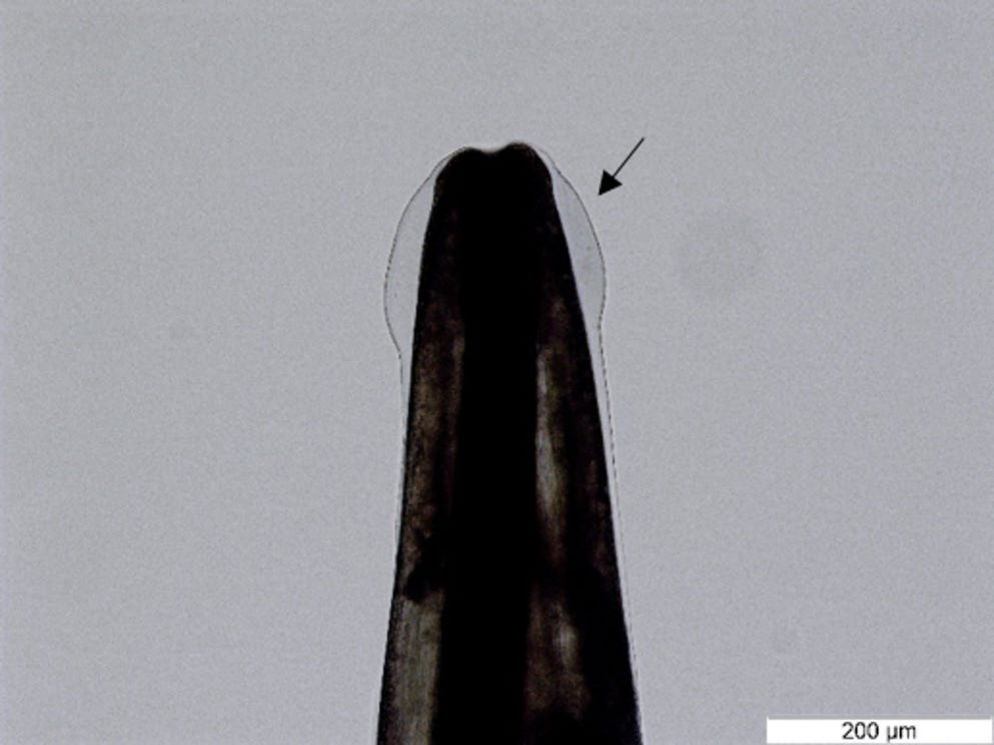
*Dictyocaulus cervi* male anterior end with the presence of cephalic vesicle (arrow)

Based on morphology of adults (Figs. 1 and 2), the nematodes had features consistent with the characteristics of *Dictyocaulus cervi*. Out of the 12 nematodes used for molecular identification, 8 were successfully sequenced and molecularly analyzed. They showed a 94.4–100% identity with *D. cervi* isolates from Poland (KM374673) and Australia (PP925798- PP925800).

**Fig. 3:**
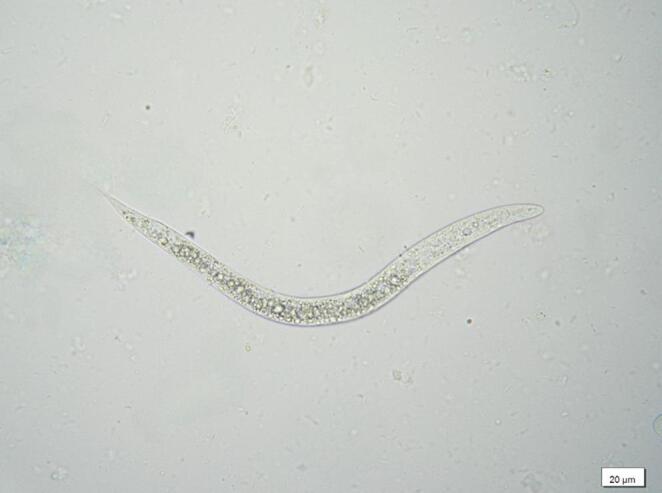
First stage larva (L1) of *Dictyocaulus cervi* collected from a Baermann sedimentation of lung tissue

The Baermann’s method performed on lung tissue showed the presence of larvae with morphological features consistent with *Dictyocaulus* spp. (Fig. 3).

During the necropsy, several macroscopic changes were noted in the four carcasses and summarized in Table 1.

**Table 1:** Summary of postmortem findings in the current study

Gross lesion	Deer 1	Deer 2	Deer 3	Deer 4
Cachexia	+	+	+	+
Pulmonary atelectasis	+	+	+	+
Presence of pulmonary parasites in the bronchi	+	+	+	+
Serous atrophy of the fat	+	+	+	+
Subcutaneous edema	+	+	+	-
Dehydration	+	+	-	-
Reactive tracheobronchial lymph nodes	+	+	-	-
Serous effusions in the pleural and pericardial cavities	-	-	+	+
Presence of adult *Haemonchus contortus* in the abomasum	+	-	-	-
Hemorrhagic content in the intestine, presence of adult parasites from genus *Trichuris*	-	+	-	-
Hydropericardium	-	+	-	-

**Fig. 4:**
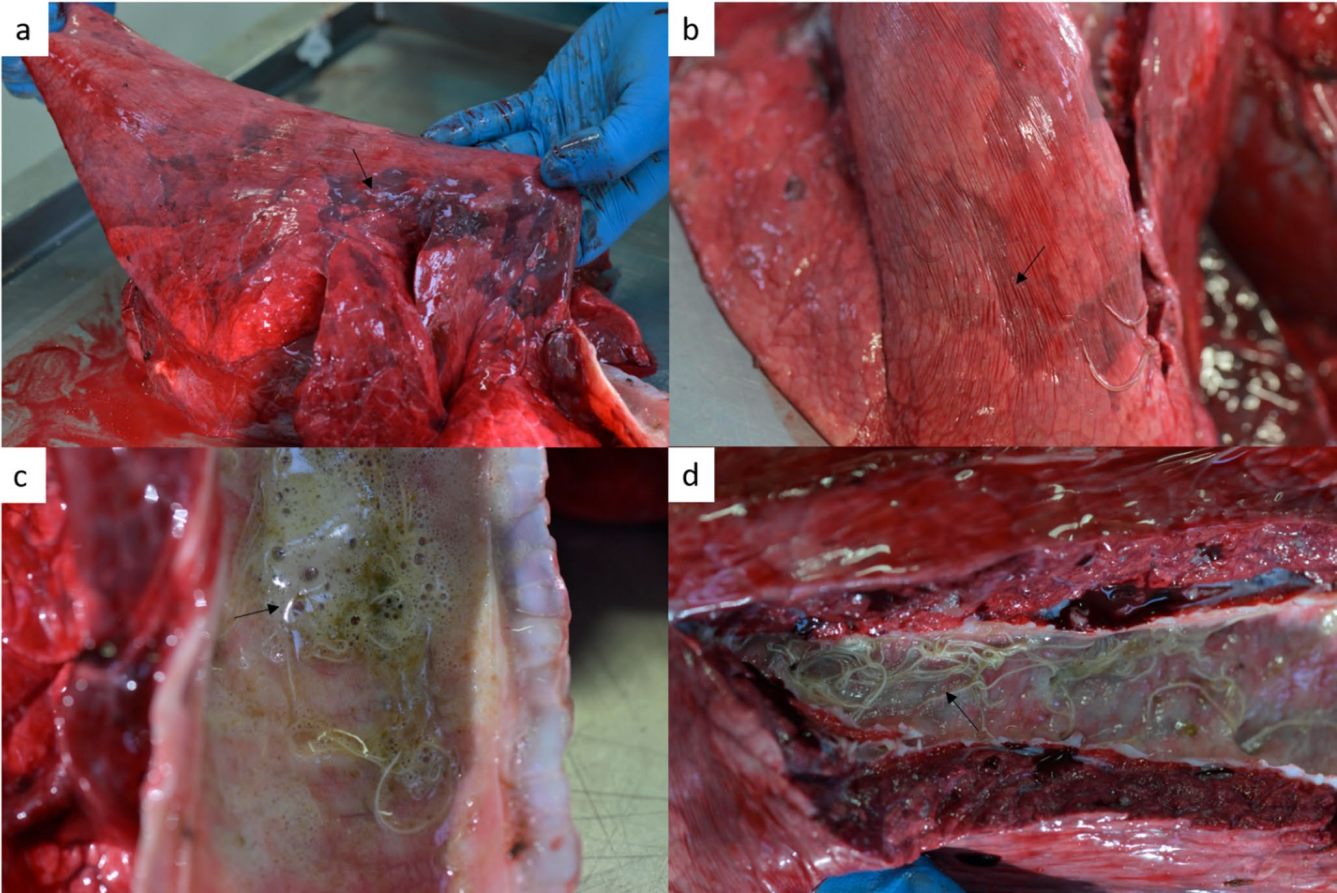
Gross findings of pulmonary changes caused by *Dictyocaulus cervi* in *Cervus elaphus*. (**a**), (**b**) Pulmonary parenchyma showing multifocal areas of obstructive atelectasis. (**c**), (**d**) The trachea and the main bronchi containing numerous nematodes admixed with edema fluid and mucus (arrows)

Macroscopically, the pulmonary parenchyma showed multifocal dark red areas of densification suggestive of atelectasis. Numerous adult nematodes were also present within the lumen of the main (primary) and lobar (secondary) bronchia admixed with edema, and mucus (Fig. 4).

**Fig. 5:**
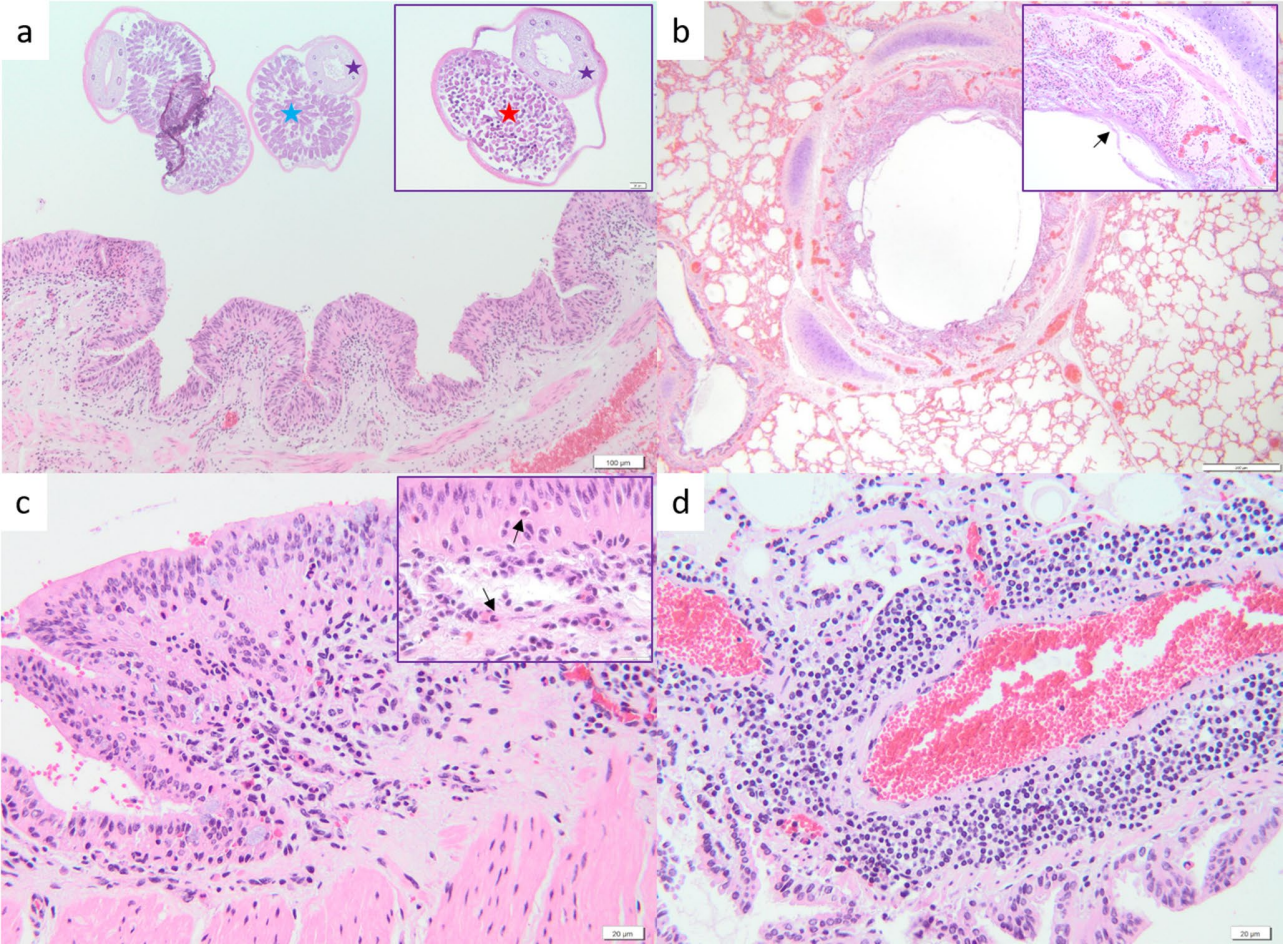
Histological aspects of the pulmonary lesions caused by *D. cervi* in a *Cervus elaphus*. (**a**) The bronchi and bronchioles are ectatic and contain cross-sectioned adult nematodes, both females and males. The nematodes show an acidophilic cuticle, digestive tract (black stars) and gonads (uterus - red star; testis - blue star). (**b**) The bronchial epithelium is covered by a moderate amount of basophilic mucus admixed with cell debris (inset). (**c**), (**d**) The bronchial and bronchiolar walls and the perivascular space are infiltrated with lymphocytes, macrophages and eosinophils (the inset, arrows)

Histologically, adult nematodes (both males and females) were observed in the bronchi, admixed with a variable amount of pale basophilic, fibrillar material, interpreted as mucus. Around the bronchi as well as the bronchioles, a moderate inflammatory infiltrate, consisting of lymphocytes, plasma cells, macrophages, and eosinophils, was identified. A similar inflammatory change was noted in the perivascular space (Fig. 5). Moderate oedema, emphysema, and congestion were noticed within the adjacent tissue. No eggs or larvae of *D. cervi* were identified within the sectioned tissues.

## Discussion

According to Caswell 2023, there are three distinct manifestations of lungworm infection in cattle: (1) acute diffuse interstitial pneumonia, usually present in calves which are exposed to larvae on contaminated pastures; (2) chronic patent infections with the presence of adult nematodes in the bronchi, representing the most common disease manifestation and (3) the “reinfection syndrome” present in adult animals that are re-infected with a large amount of larvae and can develop acute interstitial pneumonia, but the partial immune response preventing patency. The four deer in our study can be assigned to the second type of manifestation.

The presence of mucus in the airways can result from various pathological processes. However, based on the necropsy findings and macroscopic differential diagnosis, the mucus accumulation was attributed to the presence and subsequent mechanical irritation caused by adult parasites within the airways, since no macroscopic or histological lesions suggesting alternative pathologies (viral or bacterial infections) were detected. Similar changes were previously described in European bison (Cârstolovean et al. 2024), cattle (Mahmood et al. 2014), and red deer (Pyziel et al. 2017, 2018). One of the pathogenic mechanisms of *Dictyocaulus* spp. infection is represented by inflammation of bronchial mucosa, Goblet cells hyperplasia with excessive mucus production in the bronchi and bronchioles (catarrhal bronchitis and bronchiolitis). The exudate may cause obstruction of the airways, leading to pulmonary atelectasis (Lopez and Martison 2022).

In the present study, all examined red deer died as a result of respiratory failure caused by massive infection with *D. cervi.* While Pyzel et al. (2017) reported low infection intensities in free-ranging red deer from northeastern Poland, the current findings highlights that a severe parasitic infestation can contribute to mortality particularly in individuals with underlying conditions as observed in our cases with cachexia. Furthermore, in severe infestations, an excess of mucus is produced within bronchi and bronchioles which may obstruct airways (catarrhal bronchitis and bronchiolitis) leading to pulmonary atelectasis. These results highlight the need for increased epidemiological surveillance to better understand the distribution of *D. cervi* and assess its impact on wildlife health. Management practices, animal density, and stress-related factors in captivity may facilitate transmission of parasites with direct life cycle and contribute to the onset of more severe clinical manifestations (Mattiello 2009).

Microscopically, within the pulmonary tissues, only the adult stage of *D. cervi* was observed in the bronchial lumen, suggesting a patent phase of infection (Panuska 2006). The mild pathological changes within the pulmonary parenchyma further support this interpretation. Similar to the changes described by Pyzel et al. (2018), the bronchi contained a moderate amount of mucus associated with a moderate chronic inflammatory infiltrate composed of lymphocytes, plasma cells, and eosinophils. Additionally, mild to moderate inflammation was identified in the perivascular area.

Considering the high parasite load (with no standardized grading system available to assess severity, and with the formation of tangled parasites in the tracheobronchial tree indicating a heavy infection), together with severe lesions such as pulmonary edema and atelectasis, and serous fat atrophy, the most probable causes of death in all four cervids were respiratory insufficiency and cachexia.

## Conclusion

As part of preventive measures, particularly in farmed red deer, routine antiparasitic treatment should be considered, along with coproparasitological examination, to reduce the risk of clinical infection. Moreover, the distribution of *D. cervi* in Romania has not yet been studied in free-ranging cervids. Further epidemiological investigation is needed to determine its geographic distribution and to assess the potential for transmission to other ungulate hosts.

## Supplementary Information

Below is the link to the electronic supplementary material.

Supplementary Material 1

## Data Availability

Not applicable.
