# Supplementary figures and images for: Mortality associated with Dictyocaulus cervi in farmed red deer (Cervus elaphus) in Romania
Source: Parasitol Res. 2026 Feb 6;125(1):15. doi: 10.1007/s00436-026-08641-1 (PMC12876458; doi:10.1007/s00436-026-08641-1)

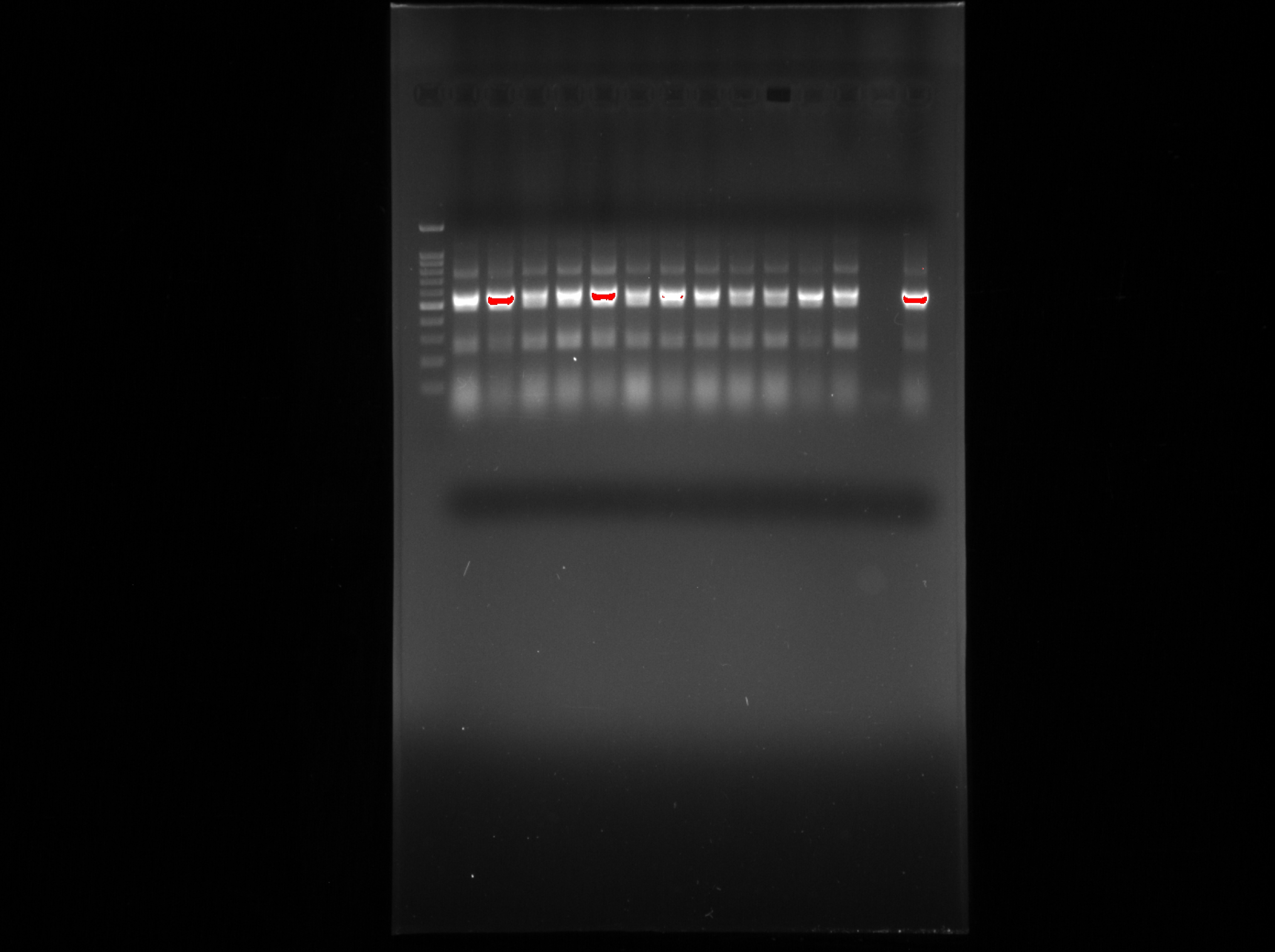

Supplement: Supplementary file 1 — Supplementary Material 1 [file 436_2026_8641_MOESM1_ESM.jpg]
